# Harnessing Lipid Polymer Hybrid Nanoparticles for Enhanced Oral Bioavailability of Thymoquinone: In Vitro and In Vivo Assessments

**DOI:** 10.3390/polym14183705

**Published:** 2022-09-06

**Authors:** Syed Sarim Imam, Sadaf Jamal Gilani, May Nasser Bin Jumah, Md. Rizwanullah, Ameeduzzafar Zafar, Mohammed Muqtader Ahmed, Sultan Alshehri

**Affiliations:** 1 Department of Pharmaceutics, College of Pharmacy, King Saud University, Riyadh 11451, Saudi Arabia; 2 Department of Basic Health Sciences, Preparatory Year, Princess Nourah bint Abdulrahman University, Riyadh 11671, Saudi Arabia; 3 Biology Department, College of Science, Princess Nourah bint Abdulrahman University, Riyadh 11671, Saudi Arabia; 4 Environment and Biomaterial Unit, Health Sciences Research Center, Princess Nourah bint Abdulrahman University, Riyadh 11671, Saudi Arabia; 5 Saudi Society for Applied Science, Princess Nourah bint Abdulrahman University, Riyadh 11671, Saudi Arabia; 6 Department of Pharmaceutics, School of Pharmaceutical Education and Research, Jamia Hamdard, New Delhi 110062, India; 7 Department of Pharmaceutics, College of Pharmacy, Jouf University, Sakaka 72341, Saudi Arabia; 8 Department of Pharmaceutics, College of Pharmacy, Prince Sattam Bin Abdulaziz University, Al-Kharj 11942, Saudi Arabia

**Keywords:** thymoquinone, lipid-polymer hybrid nanoparticles, oral bioavailability, breast cancer, cytotoxicity

## Abstract

The clinical application of phytochemicals such as thymoquinone (THQ) is restricted due to their limited aqueous solubility and oral bioavailability. Developing mucoadhesive nanocarriers to deliver these natural compounds might provide new hope to enhance their oral bioavailability. Herein, this investigation aimed to develop THQ-loaded lipid-polymer hybrid nanoparticles (THQ-LPHNPs) based on natural polymer chitosan. THQ-LPHNPs were fabricated by the nanoprecipitation technique and optimized by the 3-factor 3-level Box–Behnken design. The optimized LPHNPs represented excellent properties for ideal THQ delivery for oral administration. The optimized THQ-LPHNPs revealed the particles size (PS), polydispersity index (PDI), entrapment efficiency (%EE), and zeta potential (ZP) of <200 nm, <0.25, >85%, and >25 mV, respectively. THQ-LPHNPs represented excellent stability in the gastrointestinal milieu and storage stability in different environmental conditions. THQ-LPHNPs represented almost similar release profiles in both gastric as well as intestinal media with the initial fast release for 4 h and after that a sustained release up to 48 h. Further, the optimized THQ-LPHNPs represent excellent mucin binding efficiency (>70%). Cytotoxicity study revealed much better anti-breast cancer activity of THQ-LPHNPs compared with free THQ against MDA-MB-231 and MCF-7 breast cancer cells. Moreover, ex vivo experiments revealed more than three times higher permeation from the intestine after THQ-LPHNPs administration compared to the conventional THQ suspension. Furthermore, the THQ-LPHNPs showed 4.74-fold enhanced bioavailability after oral administration in comparison with the conventional THQ suspension. Therefore, from the above outcomes, mucoadhesive LPHNPs might be suitable nano-scale carriers for enhanced oral bioavailability and therapeutic efficacy of highly lipophilic phytochemicals such as THQ.

## 1. Introduction

In the advanced scientific era of the 21st century, breast cancer (BC) is one of the most prevalent among other solid tumors and major causes of death worldwide. As per the GLOBOCON report, ~2 million cases of BC were reported in 2018 globally, and it was expected to reach over 3 million by 2040 [1]. This deadly ailment is attributed to the uncontrolled division of malignant cells leading to the development of solid tumor in the breast or breast area [2]. The primary reason is the unhealthy lifestyle and bad environmental conditions that stagnate the estrogen levels in women, especially after menopause making them vulnerable to BC [3]. In addition, different risk factors such as frequent use of oral contraceptives, alcohol, and cigarette smoking also trigger the development of BC [1].

Since the ancient era, medicinal plants have been extensively used to treat a variety of diseases. A variety of phytochemicals represent excellent pharmacologic effects against different diseases including breast cancer [4]. In this regard, *Nigella sativa* is an annual flowering plant belonging to the Ranunculaceae family [5]. Thymoquinone (THQ) is the major constituent of *Nigella sativa* that represents a variety of therapeutic benefits such as antioxidant, anti-diabetic, anti-inflammatory, immunomodulatory, and anticancer activity [5]. However, oral delivery of THQ is still challenging due to its highly lipophilic characteristics and limited aqueous solubility. THQ represents a logP value of 2.55 and aqueous solubility of <1 mg/mL. Due to these physicochemical characteristics, the THQ represents poor dissolution in the gastrointestinal media and restricts its absorption from the small intestine which led to very limited oral bioavailability [6,7]. Therefore, an advanced delivery system is needed to improve the oral delivery of THQ.

Over the last four decades, nanotechnology-mediated drug delivery carriers (nanocarriers) gained significant attention that overcoming the challenges related to conventional drug delivery systems [8]. Therefore, scientists across the world exploited different nanocarriers to deliver THQ and improve its aqueous solubility, stability, and oral bioavailability [9,10]. Different nanocarriers have been developed to deliver THQ such as lipid-based nanocarriers [11,12], polymer-based nanocarriers [13,14], and inorganic nanocarriers [15,16] for different therapeutic applications. Following oral administration, phytochemical-encapsulated nanoparticles represent a significant increment in the surface area for absorption from the intestine due to their nanoscale size. In addition, nanoparticles encapsulate the lipophilic bioactive in the solid matrix in an amorphous state which is freely soluble in gastrointestinal fluids [17]. Furthermore, the nanocarriers show the ability to deliver the entrapped bioactive to the target site, i.e., tumor, by exploiting the enhanced permeability and retention (EPR) effect and can significantly decrease the dose-related toxicity of the encapsulated compounds [18].

Recently, lipid-polymer hybrid nanoparticles (LPHNPs) emerged as the best nanocarrier to deliver lipophilic compounds for breast cancer treatment [19]. LPHNPs are fabricated with a mixture of lipid and polymers that belongs to the generally regarded as safe (GRAS) materials approved by the FDA. LPHNPs are developed to overcome the challenges encountered in the development of both lipid and polymer-based nanocarriers [20]. LPHNPs represent a much higher loading capacity, stability in the gastrointestinal milieu, and greater dissolution rate of the encapsulated compound and enhance the absorption from the small intestine thereby higher oral bioavailability [21]. Furthermore, the LPHNPs with cationic natural polymers such as chitosan (CHS) provide excellent mucoadhesive properties to the nanoparticles [17]. CHS-based nanocarriers represent much better stability in the different pH conditions of the gastrointestinal tract (GIT) that protects the encapsulated compounds from enzymatic degradation [22]. Furthermore, the mucoadhesive characteristics of the CHS significantly enhance the residence time of the LPHNPs on the mucosa of the GIT and increase the absorption from the intestine thereby enhancing the oral bioavailability of the encapsulated compounds [23].

Based on the above facts, this study aimed to develop CHS-based mucoadhesive LPHNPs for enhanced oral delivery of THQ (abbreviated “THQ-LPHNPs”). The THQ-LPHNPs were optimized by a 3^3^-Box–Behnken design (3^3^-BBD). The optimized THQ-LPHNPs were evaluated for different pharmaceutical attributes and finally evaluated for their ability to improve the efficacy against different breast cancer cells and in vivo oral bioavailability.

## 2. Materials and Methods

### 2.1. Materials and Chemicals

Thymoquinone (THQ), Chitosan (CHS; 85% deacetylated), dialysis tube (Mol. Wt: 12–14 kDa), and Mucin protein (type II) from porcine stomach were procured from Sigma-Aldrich, St. Louis, MO, USA. Phospholipon 90G (PL-90G) was obtained as a gift sample from Lipoid, GmbH, Ludwigshafen am Rhein, Germany. Poloxamer-188 (P-188) was duly received as a gift sample from BASF, Mumbai, India. N, N-dimethylformamide was purchased from Thermo Fisher Scientific, Mumbai, India. All other chemicals and reagents were used with high purity. Animal cell lines viz. MDA-MB-231 and MCF-7 cells were purchased from National Centre for Cell Science, Pune, India.

### 2.2. Quantification of THQ in THQ-LPHNPs

THQ concentration in the present study was quantified as per our previously reported RP-HPLC technique [24]. Briefly, a binary pump-based RP-HPLC system (Waters- 2695) was attached to the UV detector (Waters 2475 Multi Lambda) and was used in the present investigation. Further, a symmetry C-18 column (Li Chospher 100; dimension 250 × 4.6 mm) was employed for the chromatographic separation at 30 °C. The mobile phase was prepared by mixing water: 2-propanol: methanol in the ratio of 50:45:5 *v*/*v*/*v*. Before the experiment, the mobile phase was degassed by sonication, and the flow rate was set at the rate of 1 mL/min. The samples were injected at a volume of 20 μL and the detection was performed at 254 nm wavelength.

### 2.3. Box–Behnken Design

In the present research, the THQ-LPHNPs were optimized by 3^3^-BBD by using Design-Expert^®^ software V-13 (State-Ease Inc., Minneapolis, MN, USA). A 3^3^-BBD was utilized to create polynomial models for optimizing the independent factors to obtain an optimized formulation. The 3^3^-BBD produces a total of 15 concentrations of the independent factors with 3 repeated center points to make the formulations. In the present investigation, the 3 independent factors namely PL-90G concentration (in mg; abbreviated as F_1_), CHS concentration (in mg; abbreviated as F_2_), and P-188 concentration (in mg; abbreviated as F_3_) were selected. All the 3 factors were varied at 3 levels namely “low”, “medium”, and “high” (Table 1). Further, the 3 responses namely particle size (PS; abbreviated as R_1_), polydispersity index (PDI; abbreviated as R_2_), and entrapment efficiency (%EE; abbreviated as R_3_ were chosen for the selection of an optimized formulation. Table 1 presents the different factors at different levels along with the selected response in BBD for the development of THQ-LPHNPs. After the development of all the 15 formulations as per the composition obtained from the 3^3^-BBD, the responses were summarized in Table 2 with their predicted values. After that, the data were fitted into the different statistical models and the best mathematical model was selected by performing a one-way analysis of variance (ANOVA). Furthermore, the overall desirability of the best-fitted model was calculated to select the optimized composition.

### 2.4. THQ-LPHNPs Production

In this research, the THQ-LPHNPs were prepared by a simple and single-step nanoprecipitation method [23,25]. For THQ-LPHNPs preparation, two separate phases viz aqueous and organic solution were prepared. The aqueous phase was prepared by the addition of CHS (50–90 mg) in 8 mL of 0.1% acetic acid solution and dissolved properly at room temperature. Then, surfactant (P-188; 75–125 mg) was added in the aqueous phase and further dissolved by gentle stirring. Subsequently, the organic solution was prepared by the addition of an accurately weighed THQ (10 mg) and lipid (PL-90G; 100–150 mg) in 2 mL of N, N-dimethylformamide and dissolved properly by gentle stirring at a speed of 850 rpm at room temperature. Then, the organic solution was dropped into the aqueous phase by using a 2.5 mL syringe at 850 rpm stirring speed. The resulting nanoparticle dispersion was continuously stirred at the same stirring speed for 3 h self-assembling of lipid and polymer into the nanoparticles. The blank LPHNPs was developed by the same procedure except for the addition of THQ. Finally, the organic solvent was removed by dialysis against double distilled water.

### 2.5. THQ-LPHNPs Characterization

#### 2.5.1. Particles Characterization

The pharmaceutical attributes such as average PS, PDI, and ZP of THQ-LPHNPs were analyzed by dynamic light scattering technique using a Zetasizer instrument (ZS 900, Malvern Instruments Ltd., Worcestershire, UK). Before measurement, the nanocarrier’s dispersion was appropriately diluted with deionized water. Finally, the scattering angle was set to 90°, and measurement was done at room temperature. The morphology of THQ-LPHNPs was visualized under a transmission electron microscope (TEM; JEM 2100 F, JEOL, Tokyo, Japan).

#### 2.5.2. Encapsulation Efficiency (%EE) and Drug Loading (%DL)

The %EE and %DL determination were based on the quantification of free THQ in the supernatant after centrifugation i.e., indirect calculation [23]. Firstly, the THQ-LPHNPs were taken in a centrifuge tube and centrifuged at 30,000× *g* for 30 min in a high-speed cooling centrifuge (Eppendorf, Stevenage SG1 2FP, UK). Then the supernatant was collected, filtered with a 0.22 µm nylon filter, and the free THQ was quantified by RP-HPLC (Waters Corporation, Milford, MA, USA) at 254 nm. Finally, the following formulae were used to calculate the %EE and %LC.

(1) $$\%\mathrm{EE}=\frac{\mathrm{Total}\;\mathrm{THQ}\;-\mathrm{Unencpsulated}\;\mathrm{THQ}\;}{\mathrm{Total}\;\mathrm{THQ}}\times 100$$

(2) $$\%\mathrm{DL}=\frac{\mathrm{Total}\;\mathrm{THQ}-\mathrm{Unencapsulated}\;\mathrm{THQ}}{\mathrm{Weight}\;\mathrm{of}\;\mathrm{LPHNPs}}\times 100$$

### 2.6. Stability Experiments

#### 2.6.1. Stability in the Gastrointestinal Milieu

The harsh gastrointestinal milieu greatly affects the pharmaceutical attributes of nanocarriers. Therefore, the stability of nanocarriers in the gastrointestinal milieu becomes important. Firstly, to prepare the simulated gastric fluid (SGF), 0.35 mL HCl and 100 NaCl were dissolved in 50 mL water. Then, 100 mg of pepsin was dissolved by gentle agitation. Finally, the pH of the fluid was adjusted by the addition of HCl to 1.2. Subsequently, to prepare the simulated intestinal fluid (SIF), 340 mg of KH_2_PO_4_ was dissolved in 50 mL of water. Then, 3.85 mL of 0.2 M NaOH and 500 mg of pancreatin were added and mixed gently. Finally, the pH of the fluid was adjusted by the addition of NaOH to 6.8 [26]. Firstly, 2 mL of THQ-LPHNPs were mixed with the 10 mL of simulated gastric fluids (SGF; pH = 1.2) and transferred in an incubator shaker for 2 h at body temperature (i.e., 37 ± 1 °C) and 100 rpm shaking speed. Subsequently, 2 mL of THQ-LPHNPs were mixed with 10 mL of simulated intestinal fluids (SIF; pH = 6.8) and transferred in an incubator shaker for 2 h at body temperature (i.e., 37 ± 1 °C) and 100 rpm shaking speed. Just after the completion of incubation time, the pharmaceutical attributes such as PS, PDI, %EE, and ZP were measured and observed for any significant variation in these parameters in comparison with initial values [27].

#### 2.6.2. Storage Stability

Before the experiment, 5 mL THQ-LPHNPs were transferred in the glass vials with 10 mL capacity. All the glass vials were then transferred into a stability chamber (Powers Scientific Inc., 150 E State St, Doylestown, PA, USA) and stored at 5 ± 1 °C, 25 ± 2 °C, and 40 ± 2 °C temperature conditions for 6 months as per the ICH guidelines [28]. After every 30th day of experiments, any changes in the pharmaceutical attributes such as PS, PDI, %EE, and ZP were measured and observed for any significant variation in these parameters in comparison with initial values.

### 2.7. THQ Release and Release Kinetics

The dissolution profile of THQ-LPHNPs was conducted in SGF (pH = 1.2) for 2 h after that in SIF (pH = 6.8) up to 48 h by the dialysis bag (Mol. Wt. 12–14 kDa) method [29]. A volume of 500 mL of gastrointestinal fluids bearing 0.5% *v*/*v* Tween 80 was used as a dissolution media. For experimenting, 5 mL of THQ-LPHNPs (~5 mg of THQ) was taken in the preactivated dialysis bag, and both ends were ligated tightly with commercial thread to avoid any leakage of the formulation. Then the dialysis bag bearing 5 mL of THQ-LPHNPs was immersed in the beaker bearing dissolution media. The beaker was kept on the magnetic stirrer at 37 ± 1°C and stirred the solution at 100 rpm speed. At each fixed time interval (i.e., 0 h, 0.5 h, 1 h, 2 h, 4 h, 6 h, 8 h, 12 h, 24 h, and 48 h), 2 mL of dissolution media was taken from the beaker and replaced with fresh media. Then the samples were diluted and filtered with a 0.4 µm nylon filter, and the dissolved quantity of THQ was quantified by the RP-HPLC technique at $\lambda_{max}$ 254 nm. Moreover, a cumulative THQ release vs. time graph was plotted to understand the release profile of THQ-LPHNPs in both dissolution media. Furthermore, the obtained results were fitted into different mathematical models to analyze the mechanism of THQ release from the solid matrix of LPHNPs [30].

### 2.8. Mucoadhesion Study

The mucoadhesive characteristics of THQ-LPHNPs was analyzed by determining the in vitro mucin binding efficiency of nanocarriers [31]. For conducting the experiment, a 0.5% *w*/*v* mucin solution was prepared in phosphate buffer solution (pH = 6.4). Then, THQ-LPHNPs was mixed with the resulting mucin solution in 1:1 and gently stirred continuously on the shaker at 37 ± 1 °C. Just after 1 h and 3 h, the samples were taken and centrifuged (Beckman Coulter Allegra X-12R, Boston, MA, USA) at 12,000× *g* at 4 °C for 10 min. After that, the supernatant was taken, and the free mucin content was quantified by Schiff colorimetric method [32]. Finally, the interaction between mucin and the optimized LPHNPs in terms of mucin binding efficiency was calculated from the following formula:

(3) $$\mathrm{Mucin}\;\mathrm{binding}\;\mathrm{efficiency}=\frac{{\mathrm{Mucin}}_{\mathrm{Total}}-{\mathrm{Mucin}}_{\mathrm{Remaining}}}{{\mathrm{Mucin}}_{\mathrm{Total}}}\times 100$$

### 2.9. Cell Culture Studies

#### 2.9.1. Cell Viability Assay

The in vitro anti-breast cancer efficacy of THQ-LPHNPs was evaluated by MTT assay in MDA-MB-231 and MCF-7 cells [33,34]. Before treatment, 96 well plates bearing DMEM media with 10% fetal bovine serum were taken and seeded with cancer cells with a density of 1 × 10^5^ cells in each plate, and then the plates are incubated for 12 h. After 90% of confluence, cells were treated with THQ-LPHNPs, free THQ, and blank LPHNPs. After that, 50 mL of MTT dye was added to each well and plate and again incubated for 3 h for the development of formazan crystals. Then, the excess media was removed and 100 mL DMSO was added. Then the plates were gently shaken on the shaker to solubilize the crystals in DMSO. Afterward, the optical density was measured for solubilized formazan with a microplate reader (BioTek, Winooski, VT, USA) at the wavelength of 570 nm. The experiment was conducted for 24 h and 48 to calculate the %cytotoxicity of THQ-LPHNPs and free THQ and the cell viability vs. concentration graph was plotted for both cells. Finally, the anticancer potential of THQ-LPHNPs and free THQ was analyzed by calculating the IC_50_ value at each time point and on each cancer cell.

#### 2.9.2. Lactate Dehydrogenase (LDH) Assay

The LDH assay was performed to evaluate the membrane integrity of MDA-MB-231 and MCF-7 cells after treatment with the nanoparticles and compared the results with free THQ. This study was performed as per the protocol provided manufacturer of in vitro toxicology assay kit (Sigma-Aldrich, USA). Briefly, both breast cancer cells at a density of 1 × 10^5^ were treated with the respective IC_50_ concentrations of THQ and THQ-LPHNPs for 24 h and 48 h. Then, 100 µL of cell-free supernatant was taken and transferred into a new 96-well plate. Subsequently, 100 µL of LDH assay reaction mixture was properly mixed with the cells in each well and incubated for 3 h [35]. After incubation, the optical density (OD) was measured using a microplate reader (BioTek, Winooski, VT, USA) at a wavelength of 490 nm.

#### 2.9.3. Morphological Examination of Treated Cells

The change in morphology of cancer cells after treatment with the free drug and nanocarrier was analyzed by phase-contrast microscopy [36]. MDA-MB-231 and MCF-7 cells (1 × 10^5^ cells) were cultured in 6-well microplates and incubated overnight. After confluence, both cells were treated with THQ and THQ-LPHNPs at their IC_50_ dose calculated from the MTT assay and incubated for 24 and 72 h, respectively. After completion of the treatment period, the morphological examination was performed with the help of an inverted microscope (Nikon TE200, Minato ku, Japan).

### 2.10. Ex Vivo THQ Permeation Study

The drug permeation from the small intestine after oral administration of THQ-LPHNPs was analyzed by using a rat intestine as per the reported protocol [37]. Before the experiment, rats were fasted for 12 h and sacrificed by cervical dislocation and the small intestine (5 cm long) was taken. Then the intestine was washed with Tyrode solution to remove any extra food residue. Subsequently, 2 mL of THQ-LPHNPs (~2 mg THQ) was transferred into the intestinal sac and both ends were ligated tightly by using commercial thread. Then the intestine bearing 2 mL of THQ-LPHNPs was immersed in the beaker bearing dissolution media. Throughout the experiment, the beaker was continuously aerated with an aerator. The beaker was kept on the magnetic stirrer at 37 ± 1 °C and continuously stirred the solution at 50 rpm. At the time interval of 15 min for 180 min, 2 mL of aliquots were taken from the beaker and replaced with fresh media. Then the samples were diluted and filtered with a 0.4 µm nylon filter, and the permeated quantity of THQ was quantified by the RP-HPLC technique at 254 nm $\lambda_{max}$. The intestinal permeation of THQ suspension was further conducted by the same procedure and the obtained results were compared to analyze the permeation ability of the formulations. Finally, from the observed data, the flux, apparent permeability coefficient (APC), and enhancement ratio (ER), were calculated by applying the following equations.

(4) $$\mathrm{APC}=\frac{\mathrm{Flux}}{\mathrm{Sac}\;\mathrm{area}\times {\mathrm{THQ}}_{\mathrm{Total}}}$$

(5) $$\mathrm{ER}=\frac{{\mathrm{APC}}_{\mathrm{THQ}-\mathrm{LPHNPs}}}{{\mathrm{APC}}_{\mathrm{THQ}-\mathrm{Suspension}}}$$

### 2.11. Measurement of Permeation Depth

To determine the permeation depth in the layers of the small intestine, a similar procedure as described in Section 2.9.1. The depth was analyzed by a confocal laser scanning microscope (CLSM) [38]. Briefly, 0.03% *w*/*v* Rhodamine B (RhB) loaded LPHNPs was prepared, and 2 mL was transferred into the intestinal sac and both ends were ligated tightly by using commercial thread. Then the intestine containing 2 mL of RhB-LPHNPs was immersed in the dissolution media. The media was continuously aerated with an aerator and the temperature was fixed at 37 ± 1 °C with continuous stirring of 50 rpm for 3 h. After that, the intestine was removed from the beaker and excess dye present in the small intestine was removed by washing with Tyrode solution. Then, the intestine was cut longitudinally with a surgical scissor and fixed on the glass slide. Finally, the depth of the dye was measured under CLSM (Carl Zeiss Microscopy, New York, NY, USA) at 514 nm fluorescence excitation. The penetration depth for THQ suspension was observed by following the same procedure and the observed ‘*z*-axis was compared with the ‘*z*-axis’ of RhB-LPHNPs treated slides to analyze the permeation ability of the formulations.

### 2.12. Relative Bioavailability Study

A single dose (20 mg/kg) pharmacokinetic study in Wistar rats was conducted to analyze the potential of THQ-LPHNP in the enhancement of bioavailability after oral administration and the results were compared with the results of conventional THQ suspension. Before the experiment, the rats were fasted overnight with free access to tap water. Two groups of 6 animals are made and labeled as “group I” and “group II”. Rats from group I and group II were fed with THQ-LPHNPs and THQ-Suspension (THQ suspended in 0.25% *w*/*v* carboxymethylcellulose sodium solution) orally [39]. At predetermined intervals of 0.5, 1 2, 4, 6, 8, 12, 24, and 48 h after administration, 0.5 mL of blood was collected from the tail vein and transferred into EDTA-coated centrifuge tubes. Then the blood samples were centrifuged at 8000 rpm for 5 min and the supernatant (i.e., plasma) was stored at −20 °C until analysis. To quantify the concentration of THQ in plasma, the supernatant was taken, diluted with mobile phase, and quantified by the RP-HPLC technique. In the end, various biopharmaceutical attributes are calculated from the plasma THQ concentration vs. time profiles with the help of WinNonlin^®^ software (Apex, NC, USA) for both THQ-LPHNPs and THQ-Suspension and the results were compared.

### 2.13. Statistical Analysis

All the observed data are reported as average ± standard deviation and the statistical analysis of the observed data was done with the help of GraphPad Prism version 8.0. by one-way ANOVA followed by Student’s ‘*t*’ test. The data were considered significant only when the *p*-value was <0.05.

## 3. Results and Discussion

### 3.1. THQ-LPHNPs Optimization by 3^3^-BBD

THQ-LPHNPs were prepared as per the suggested composition obtained from the 3^3^-BBD and their responses are fitted in the design as represented in Table 2. After that, the three mathematical models viz linear, two-factor interaction, and quadratic models were assessed to analyze the best-fit model to obtain a relation between the factors and responses. The mathematical model that provides maximum predicted and adjusted R^2^ was the best-fit mathematical model (Table 3). ANOVA analysis has been conducted to confirm the significant term of the best-fitted model. The lack of fit test was conducted, and the model was considered when the value is found to be >0.1, i.e., non-significant. Further, the efficacy of each factor was considered significant when the effect was not equal to 0 and their *p*-value was <0.05. For each response, a secondary polynomial equation was generated that represents a relationship between factors and responses.

According to the results, the quadratic model represented the maximum adjusted and predicted R^2^ for each response (Table 3). The lack of fit value for each response was <0.05. Further, the actual and predicted values were found to be close to each other. Finally, different statistical graphs, i.e., predicted vs. actual, 3D surface, contour, and perturbation plots were generated to analyze the effect of each factor on each response.

#### 3.1.1. Influence of Factors on R_1_

The measured PS of the investigated THQ-LPHNPs are summarized in Table 2. The PS of the THQ-LPHNPs varied from 123.79 nm to 246.53 nm. The model terms were significant with *p* = 0.0344 and the lack of fit was non-significant with *p* = 0.0945. Therefore, these results confirmed the adequacy of the quadratic model. The obtained polynomial equation from the design for R_1_ (PS) is represented as follows:

PS (R_1_) = +180.58 + 36.48F_1_ + 25.58F_2_ − 5.69C + 0.305F_1_F_2_ − 1.60F_1_F_3_ + 0.17F_2_F_3_ + 1.89F_1_^2^ + 2.38F_2_^2^ + 1.15F_3_^2^ (6)

**Figure 1:**
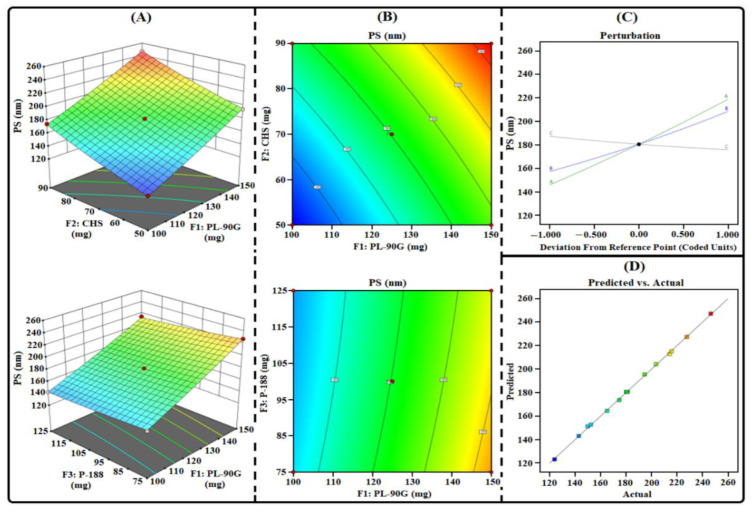
Different response plots (**A**) 3D surface, (**B**) contour, (**C**) predicted vs. actual, and (**D**) perturbation illustrating the influence of factors (F_1_, F_2_, and F_3_) on PS (abbreviated as R_1_) of THQ-LPHNPs.

The statistical plots (Figure 1) and polynomial Equation (6) suggested that each factor significantly influences the response (R_1_). The PL-90G (abbreviated as “F_1_”) and CHS (abbreviated as “F_2_”) produced a positive influence on R_1_. A gradual increase in the quantity of F_1_ from 100 mg to 150 mg significantly enhances the interfacial tension between the aqueous and organic phase which results in the coalescence of PL-90G and produced large-sized particles [28]. Subsequently, a gradual increase in the quantity of F_2_ from 50 mg to 90 mg significantly enhances the viscosity of the organic phase which produced large-sized particles [40,41]. Conversely, the P-188 (abbreviated as “F_3_”) produced a negative influence on R_1_. A gradual increase in the concentration of F_3_ from 75 mg to 125 mg significantly reduced the interfacial tension between the two phases and increase the emulsification process and produced small-sized particles [29].

#### 3.1.2. Influence of Factors on R_2_

The measured PDI of the investigated THQ-LPHNPs are summarized in Table 2. The PDI of the THQ-LPHNPs varied from 0.126 to 0.423 nm. The model terms were significant with *p* = 0.0036 and the lack of fit was non-significant with *p* = 0.0862. Therefore, these results confirmed the adequacy of the quadratic model. The obtained polynomial equation from the design for R_2_ (PDI) is represented as follows:

R_2_ (PDI) = +0.2227 + 0.0896F_1_ + 0.066F_2_ − 0.0086F_3_ + 0.0112F_1_F_2_ + 0.001F_1_F_3_ + 0.0057F_2_F_3_ +0.0234F_1_^2^ + 0.0172F_2_^2^ + 0.0204F_3_^2^ (7)

**Figure 2:**
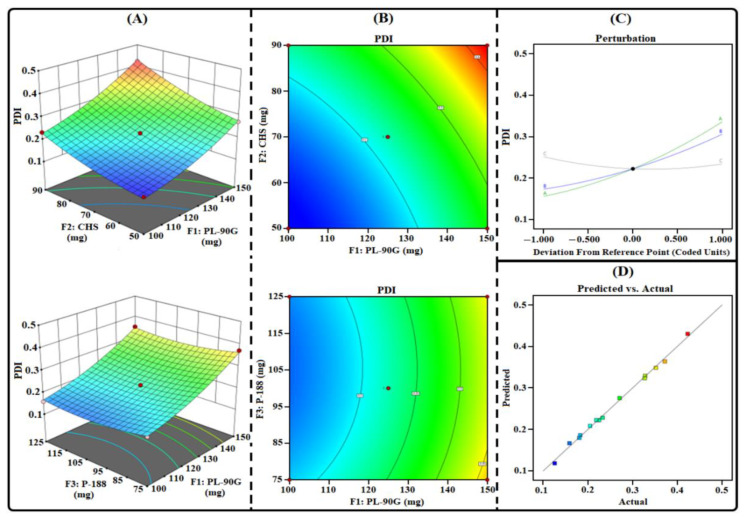
Different response plots (**A**) 3D surface, (**B**) contour, (**C**) predicted vs. actual, and (**D**) perturbation illustrating the influence of factors (F_1_, F_2_, and F_3_) on PDI (abbreviated as R_2_) of THQ-LPHNPs.

The statistical plots (Figure 2) and polynomial Equation (7) suggested that each factor significantly influences the response R_2_. The PL-90G (abbreviated as “F_1_”) and CHS (abbreviated as “F_2_”) produced a positive influence on R_2_. A gradual increase in the quantity of F_1_ from 100 mg to 150 mg significantly enhances the viscosity of the organic phase which gradually enhanced the heterogeneity between the particles that produced nanocarrier with high PDI [42]. Subsequently, a gradual increase in the quantity of F_2_ from 50 mg to 90 mg produced a coarse dispersion of nanocarriers due to a lack of energy and produced nanocarriers with high PDI [43]. Conversely, the P-188 (abbreviated as “F_3_”) produced a negative influence on R_1_. A gradual increase in the concentration of F_3_ from 75 mg to 125 mg significantly reduced the interfacial tension between the two phases and increased the emulsification process and produced small-sized particles with excellent homogeneity [25].

#### 3.1.3. Influence of Factors on R_3_

The measured %EE of the investigated THQ-LPHNPs are summarized in Table 2. The %EE of the THQ-LPHNPs varied from 69.86% to 97.65%. The model terms were significant with *p* = 0.0049 and the lack of fit was non-significant with *p* = 0.1731. Therefore, these results confirmed the adequacy of the quadratic model. The obtained polynomial equation from the design for R_2_ (PDI) is represented as follows:

R_3_ (EE) = +84.48 +8.13F_1_ + 5.63F_2_ + 1.65F_3_ + 0.1875F_1_F_2_ − 0.2775F_1_F_3_ − 0.0525F_2_F_3_ − 0.3604F_1_^2^ − 0.5554F_2_^2^ − 0.3304F_3_^2^ (8)

**Figure 3:**
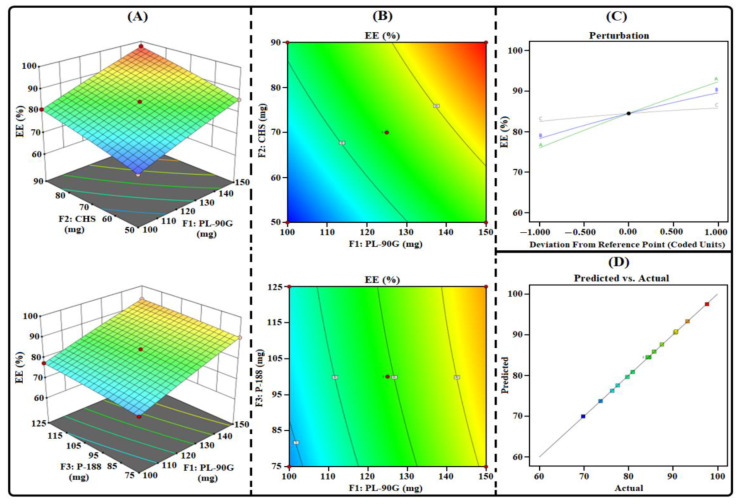
Different response plots (**A**) 3D surface, (**B**) contour, (**C**) predicted vs. actual, and (**D**) perturbation illustrating the influence of factors (F_1_, F_2_, and F_3_) on %EE (abbreviated as R_3_) of THQ-LPHNPs.

The statistical plots (Figure 3) and polynomial Equation (8) suggested that each factor significantly influences the R_3_. All three factors viz PL-90G (abbreviated as “F_1_”) and CHS (abbreviated as “F_2_”) and P-188 (abbreviated as “F_3_”) produced a positive influence on R_3_. A gradual increase in the quantity of F_1_ from 100 mg to 150 mg significantly enhances the viscosity of the organic phase resulting in rapid solidification at room temperature. Rapid solidification of PL-90G at room temperature inhibits the leakage of THQ from the outer layers of nanocarriers and produced nanocarriers with a high %EE [44]. Similarly, a gradual increase in the quantity of F_2_ from 50 mg to 90 mg significantly enhances the space for encapsulation of THQ in the amorphous state and produced nanocarriers with a high %EE [45]. Subsequently, a gradual increase in the concentration of F_3_ from 75 mg to 125 mg significantly enhances the emulsification process and produced nanocarriers with a high %EE [46].

#### 3.1.4. Selection of Optimized Composition

The optimized composition for the development of the optimized THQ-LPHNPs was chosen by applying constraints on the PS, PDI, and %EE (Table 2). After that, the software suggested an optimized composition with overall desirability of 0.915. According to the criteria, the concentration of the independent factors suggested by the 3^3^-BBD for the optimized formulation was 125 mg of PL-90G, 70 mg of CHS, and 100 mg of P-188 respectively. The optimized THQ-LPHNPs showed the PS of 179.63 ± 4.77 nm, PDI of 0.21 ± 0.01, and EE of 85.49 ± 3.73%. This optimized THQ-LPHNPs was further characterized for different parameters.

### 3.2. THQ-LPHNPs Characterization

#### 3.2.1. Particles Characterization

**Figure 4:**
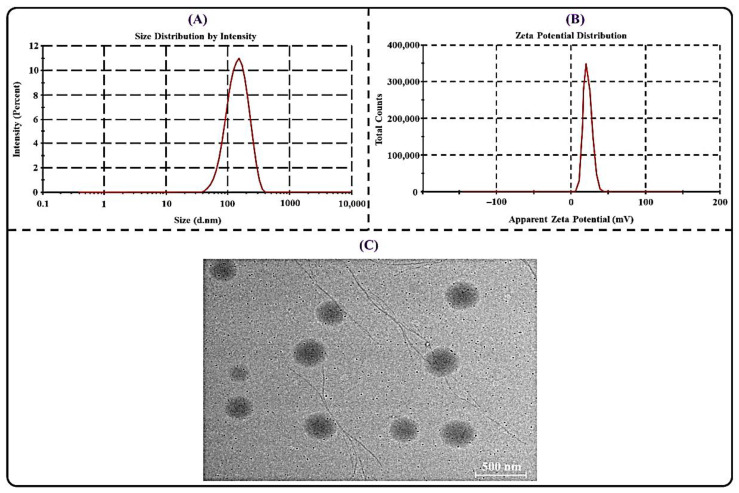
Image illustrating (**A**) particle size and size distribution, (**B**) zeta potential distribution, and (**C**) TEM micrograph of THQ-LPHNPs.

The PS of the nanocarrier plays a significant role in an ideal oral delivery. The PS should be small enough so that it can represent a greater surface area to absorb the encapsulated drugs [47]. In the present research, the average PS of the optimized THQ-LPHNPs was observed to be 179.63 ± 4.77 nm as depicted in Figure 4A. The PDI of a nanocarrier system denotes the homogeneity among the particles. Therefore, the PDI should be low for an excellent nanocarrier system to deliver the drug to improve oral bioavailability. The PDI value of <0.3 represents an excellent homogeneity among the particles [48]. In this research, the PDI value was found to be 0.21 ± 0.01 for the optimized THQ-LPHNPs. Thus, it can be inferred that our developed nanocarriers represent excellent homogeneity. ZP for ideal nanocarriers should be high to maintain its colloidal stability under different environmental conditions. A high surface charge on the particles leads to repulsion among each other and significantly reduces aggregation [49]. The ZP image for the optimized THQ-LPHNPs is depicted in Figure 4B and the value was observed to be +26.52 ± 2.21 mV. A positive surface charge on the THQ-LPHNPs ascribed to the presence of CHS on the outer layer of the nanocarrier. The positively charged nanocarrier is always advantageous because it significantly interacts with the negatively charged mucosal membrane. The electrostatic interaction between the nanocarrier and mucosal membrane results in the prolonged residence in the small intestine resulting in higher absorption of encapsulated drug [50]. The TEM analysis was conducted to examine the shape of THQ-LPHNPs and the obtained image represented in Figure 4C. TEM micrograph of the optimized THQ-LPHNPs showed distinct spherical particles.

#### 3.2.2. %EE and %DL

The %EE and %DL of the nanocarrier should be high enough to obtain desired results. In the present research, the optimized THQ-LPHNPs showed the %EE and %DL of 85.49 ± 3.73%, and 8.34 ± 0.67%, respectively. Therefore, an optimum level of %EE and %LC was observed for THQ-LPHNPs and it can be inferred that nanocarrier might be an ideal delivery system for THQ.

### 3.3. Stability Results

#### 3.3.1. Stability in the Gastrointestinal Milieu

The developed LPHNPs should be stable in the hostile GIT environment. Since the LPHNPs were developed for oral administration, therefore, the LPHNPs should maintain their pharmaceutical attributes in the hostile gastrointestinal environment and protect the encapsulated THQ from enzymatic degradation after oral administration. The results of all the selected pharmaceutical attributes in SFG and SIF are represented in Table 4. The results suggested that THQ-LPHNPs were found stable in both fluids and showed only insignificant (*p* > 0.05) variations in their evaluated parameters. After 2 h of incubation in the SGF, the PS, PDI, %EE, and ZP of the THQ-LPHNPs were observed to be 193.31 ± 6.44 nm, 0.27 ± 0.01, 78.54 ± 3.57 %, and +21.18 ± 2.83 mV, respectively. On the other hand, after 6 h of incubation in the SIF, the PS, PDI, %EE, and ZP of the THQ-LPHNPs were observed to be 187.82 ± 5.39 nm, 0.24 ± 0.01, 81.46 ± 3.39%, and +23.53 ± 1.94, respectively. Therefore, it can be inferred that our developed THQ-LPHNPs are stable in the gastrointestinal fluids and maintain their properties in the hostile gastrointestinal environment.

#### 3.3.2. Storage Stability

**Figure 5:**
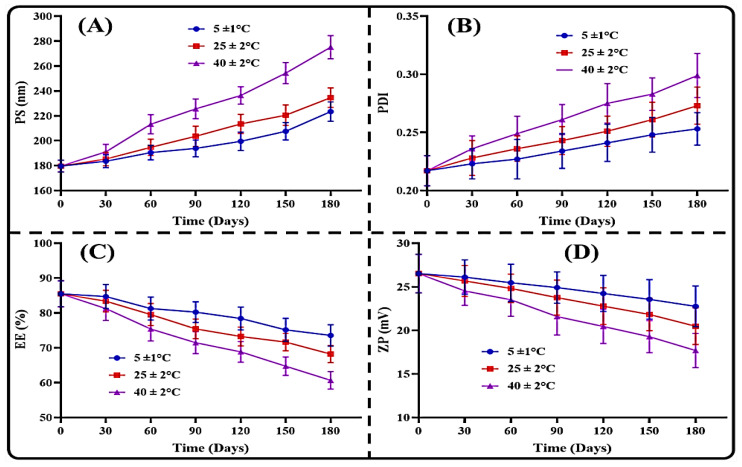
The effect of different environmental conditions at different time points on the (**A**) PS, (**B**) PDI, (**C**) %EE, and (**D**) ZP of THQ THQ-LPHNPs. Data represented as mean ± SD, *n* = 3.

The storage stability evaluation was performed in different storage and temperature conditions for 180 days. The results of all the different storage temperature conditions are depicted in Figure 5. The results suggested that the THQ-LPHNPs revealed excellent stability at 4 ± 1 °C with insignificant (*p* > 0.05) variations in the results after 180 days of storage. Subsequently, the THQ-LPHNPs revealed only minor changes at 25 ± 2 °C temperature, and results were found within the acceptable limits. At a temperature of 40 ± 2 °C, the THQ-LPHNPs revealed significant (*p* < 0.05) changes and suggested instability at a higher temperature. A significant change at 40 ± 2 °C was found due to the degradation of PL-90G and the formation of conglomerates takes place. Therefore, it can be inferred that the THQ-LPHNPs should not be stored at higher temperature to prevent the degradation.

### 3.4. THQ Release and Release Kinetics

**Figure 6:**
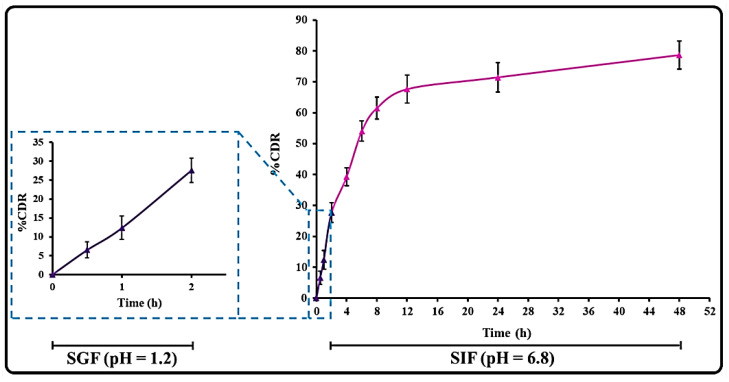
Image representing the release profiles of THQ from the THQ-LPHNPs in SGF (pH = 1.2) for the initial 2 h followed by in SIF (pH = 6.8) up to 48 h. Data represented as mean ± SD, *n* = 3.

The THQ release profiles of the optimized THQ-LPHNP were performed in SGF for 2 h, as well as SIF for up to 48 h (Figure 6). For an initial 2 h in SGF, the THQ-LPHNPs demonstrate 27.63 ± 3.29%, and after that in SIF from 2 to 48 h, the THQ release was observed to be 78.65 ± 4.21%. Initially, a fast release of THQ from LPHNPs was observed due to the presence of the drug on the surface of LPHNPs and the rapid diffusion of encapsulated drugs from the peripheral layers of the solid matrix. Moreover, the small size of LPHNPs further contributes to the fast release of drugs from the solid matrix [51]. After 4 h of study to 48 h, the THQ was released in a controlled manner due to the slow and steady dissolution of the encapsulated drug from the inner solid matrix of LPHNPs. In addition, the slow degradation properties of CHS in the gastrointestinal fluids are also a reason behind the controlled release of drugs from LPHNPs [52].

After the experiment, the obtained results were fitted into different mathematical models to analyze the mechanism of THQ release from the solid matrix of LPHNPs. The obtained results are summarized in Table 5. According to the observed results, the Korsmeyer–Peppas model showed the highest R^2^ (0.8736). Thus, the Korsmeyer–Peppas model was chosen as the best model to explain the mechanism of drug diffusion from the nanocarrier [53]. After that, the release exponent i.e., “*n*” for the selected model was calculated from the slope of the mathematical model. The value of the exponent “*n*” from the selected mathematical model was calculated and found to be 0.2397 (<0.5) and it can be inferred that the THQ release mechanism was found to be “Fickian diffusion”. A Fickian diffusion-based THQ release mechanism from LPHNPs represents that the developed formulation releases the loaded drug for the nanocarrier as per Fick’s law [54].

### 3.5. Mucoadhesion Study

CHS-based mucoadhesive nanocarriers significantly interact with mucins which are the protein component of mucus that present on the epithelium. Thus, the mucin binding efficiency of the optimized THQ-LPHNPs was evaluated at 1 h and 3 h to analyze the bio- adhesive capacity. The mucin binding efficiency for the optimized THQ-LPHNPs after 1 and 3 h was observed to be 57.63 ± 2.77% and 78.85 ± 4.39%, respectively. A greater than 50% of mucin binding efficiency represented a strong interaction between the positively charged THQ-LPHNPs and negatively charged mucin. A strong electrostatic interaction was observed due to the strong cationic characteristics of CHS. Mucoadhesive nanoparticles adhere to the mucous membrane of the GIT and reside on the epithelium for a prolonged period which helps in the greater absorption of the encapsulated compounds in the systemic circulation [55].

### 3.6. Cell Culture Studies

#### 3.6.1. Cell Viability Assay

**Figure 7:**
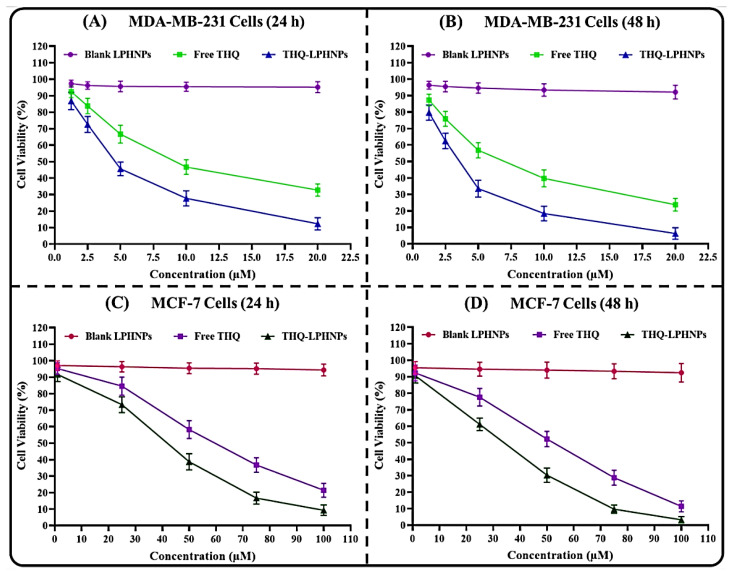
Image representing the cytotoxicity profiles of THQ-LPHNPs, free THQ, and blank LPHNPs against (**A**) MDA-MB-231 cells after 24 h, (**B**) MDA-MB-231 cells after 48 h, (**C**) MCF-7 cells after 24 h, and (**D**) MCF-7 cells after 48 h, respectively. Data represented as mean ± SD, *n* = 3.

**Figure 8:**
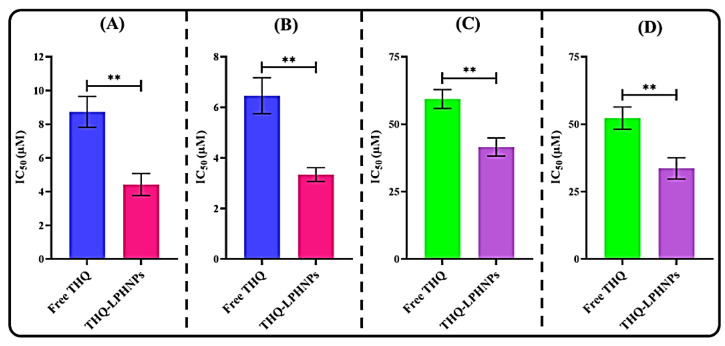
Image representing the IC_50_ values of THQ-LPHNPs and free THQ against (**A**) MDA-MB-231 cells after 24 h, (**B**) MDA-MB-231 cells after 48 h, (**C**) MCF-7 cells after 24 h, and (**D**) MCF-7 cells after 48 h. ** *p* < 0.01 vs. free THQ. Data represented as mean ± SD, *n* = 3.

From the MTT assay, the comparative cytotoxicity of THQ-LPHNPs and free THQ against MDA-MB-231 and MCF-7 cell lines was analyzed at 24 h and 48 h, respectively. The cells were also treated with blank LPHNPs to understand the effect of blank nanoparticles against both cell lines. The dose-dependent as well as time-dependent cytotoxicity profiles are depicted in Figure 7. Furthermore, the IC_50_ values at each time point against both cancer cells are represented in Figure 8. Firstly, the blank PLHNPs exhibited negligible cytotoxicity against the tested cell lines, and it was concluded that the PLHNPs were safe and compatible for oral delivery. On the other hand, the optimized THQ-LPHNPs, as well as free THQ, showed dose and time-dependent cytotoxicity in both cell lines. The optimized THQ-LPHNPs revealed a much better (*p* < 0.05) effect in comparison to free THQ. After 24 h of treatment, the optimized THQ-LPHNPs and free THQ exhibited an IC_50_ value of 4.42 ± 0.65 µM and 8.73 ± 0.92 µM, respectively, against MDA-MB-231 cells (Figure 8A), while after 48 h, they exhibited an IC_50_ value of 3.34 ± 0.27 µM and 6.46 ± 0.71 µM, respectively (Figure 8B). At 24 h, MCF-7 cells treated with the optimized THQ-LPHNPs and free THQ showed an IC_50_ value of 41.56 ± 3.35 µM and 59.37 ± 3.52 µM, respectively (Figure 8C). While after 48 h, the IC_50_ value was found to be 33.63 ± 3.95 µM and 52.28 ± 4.12 µM, respectively (Figure 8D). As per the results, better cytotoxicity was achieved after treatment with the optimized THQ-LPHNPs against both cell lines. Better cytotoxicity with THQ-LPHNPs was achieved due to the small size of the nanocarrier which produces a higher surface area for internalization into the cancer cells [56]. The tumor endothelial cells are characterized by a gap of 50–500 nm that causes a higher permeation and accumulation of small-sized (<500 nm) particles by the EPR [57]. Furthermore, a controlled release of THQ from the solid matrix of a nanocarrier allows continuous exposure of encapsulated THQ to the cancer cells, resulting in better cytotoxicity [58].

#### 3.6.2. Lactate Dehydrogenase (LDH) Assay

**Figure 9:**
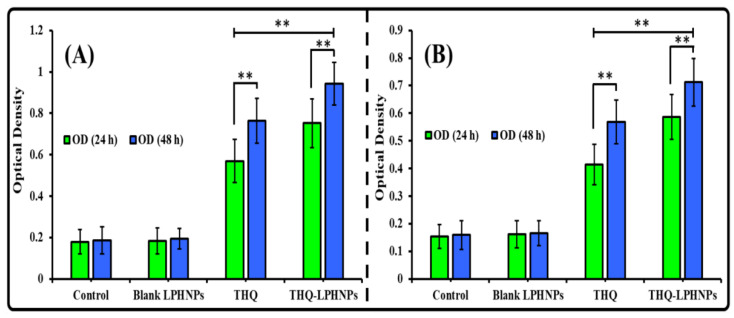
The calculated optical density after treatment with THQ and THQ-LPHNPs after 24 h and 48 h against (**A**) MDA-MB-231 and (**B**) MCF-7 cells. ** *p* < 0.01 vs. free THQ. Data represented as mean ± SD, *n* = 3.

The results of the LDH assay support the results of the MTT assay and data shown in Figure 9. As per the results, the OD value for the control group (i.e., non-treated) and blank LPHNPs was found to be non-significant to each other at 24 h as well as 48 h. Therefore, it can be inferred that the blank LPHNPs revealed insignificant cytotoxicity and are safe for drug delivery. Whereas, after treatment with THQ and THQ-LPHNPs, significant LDH was released. After 24 h, MDA-MB-231 cells treated with the optimized THQ-LPHNPs and free THQ showed an OD value of 0.752 ± 0.11 and 0.569 ± 0.11, respectively. Whereas after 48 h, the optimized THQ-LPHNPs and free THQ showed an OD value of 0.943 ± 0.13 and 0.764 ± 0.11, respectively. In the case of MCF-7 cells, the optimized THQ-LPHNPs and free THQ showed an OD of 0.587 ± 0.08 and 0.414 ± 0.07, respectively, while after 48 h of treatment, the optimized THQ-LPHNPs and free THQ showed an OD value of 0.712 ± 0.087 and 0.568 ± 0.079, respectively. Therefore, a dose and time-dependent LDH release from both cancer cells was achieved. Furthermore, the optimized THQ-LPHNPs revealed significantly enhanced LDH release from both cancer cells compared with the free drug. The higher LDH release after treatment with the optimized THQ-LPHNPs ascribed to the controlled release of THQ from the solid matrix of the nanocarrier, which causes continuous exposure of encapsulated THQ to the cancer cells. Similar results were reported by Bhattacharya et al. [59] after treatment with THQ encapsulated hyaluronic acid modified Pluronic^®^-based nanocarrier.

#### 3.6.3. Morphological Examination of Treated Cells

**Figure 10:**
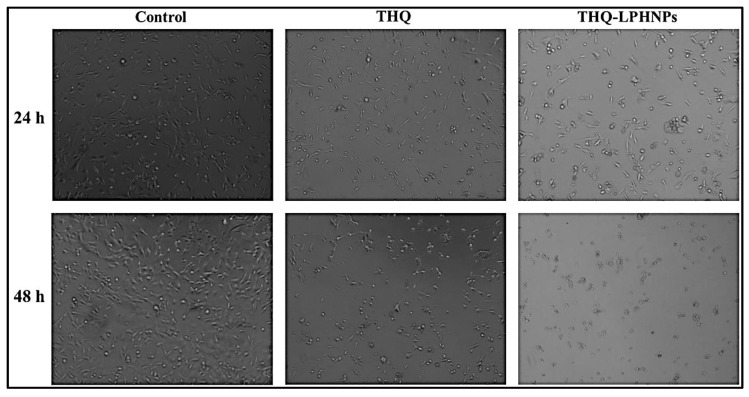
Photomicrographs of THQ and THQ-LPHNPs treated MDA-MB-231 cells after 24 h and 48 h of treatment respectively.

**Figure 11:**
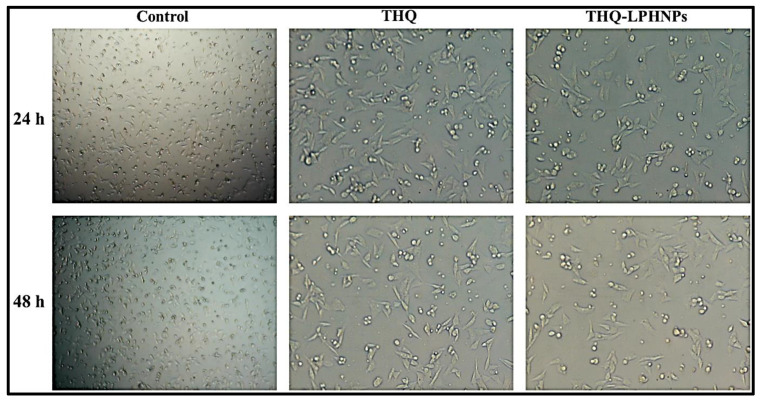
Photomicrographs of THQ and THQ-LPHNPs treated MCF-7 cells after 24 h and 48 h of treatment respectively.

The morphological examination of the cancer cells was conducted to validate the cytotoxicity results. The micrographs of MDA-MB-231 and MCF-7 cells after treatment with free THQ and THQ-LPHNPs at their IC_50_ dose at 24 and 48 h are depicted in Figure 10 and Figure 11, respectively. Both MDA-MB-231 and MCF-7 cells retained their normal polygonal shape with an intact monolayer appearance. Whereas, after treatment with both THQ and THQ-LPHNPs, the majority of the cells showed morphological variations such as cellular shrinkage, membrane blebbing, and apoptotic bodies. Furthermore, time-dependent reduction in cell number and poor adherence among the cells were observed in both cancer cells. Therefore, this investigation further supports the cytotoxic effect of THQ and THQ-LPHNPs on both cancer cells as observed in the cell viability assay.

### 3.7. Ex Vivo THQ Permeation Study

**Figure 12:**
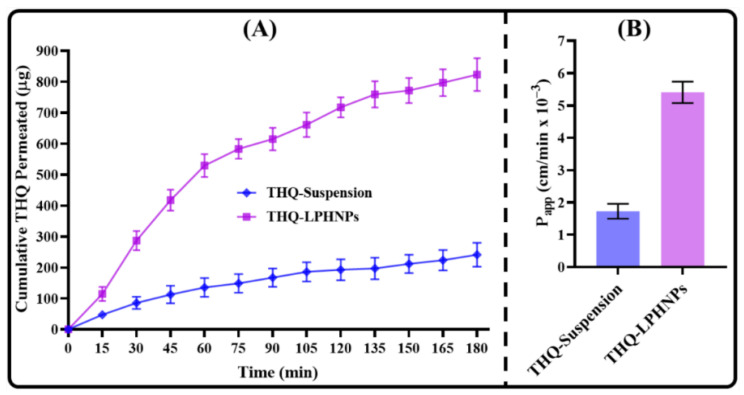
Image representing (**A**) comparative intestinal permeation profiles of THQ-LPHNPs and free THQ suspension and (**B**) apparent permeability coefficient of THQ-LPHNPs and free THQ suspension. Data represented as *n* = 3, mean ± SD.

The ex vivo intestinal permeation study across small intestine of Wistar rats was conducted for THQ-LPHNPs and THQ suspension. The cumulative THQ intestinal permeation profiles and APC of THQ-LPHNPs and THQ suspension are depicted in Figure 12. In this investigation, the optimized THQ-LPHNPs and THQ suspension exhibited intestinal permeation of 823.43 ± 53.17 μg/cm^2^ and 241.47 ± 38.53 μg/cm^2^, respectively (Figure 12A). Thus, THQ-LPHNPs showed more than 3 times (*p* < 0.05) permeation across the small intestine as compared to THQ suspension. In addition, the optimized THQ-LPHNPs and conventional THQ suspension revealed the APC of 5.41 × 10^−3^ cm/min and 1.73 × 10^−3^ cm/min, respectively (Figure 12B). Thus, THQ-LPHNPs showed almost 3 folds higher ER in comparison with THQ suspension. A much better intestinal permeability with THQ-LPHNPs was achieved due to the small particle size (<200 nm) that provides a higher surface area for absorption from the small intestine by paracellular transport. In addition, excellent mucoadhesive characteristics of LPHNPs provide much higher residence time on the intestinal mucosa and also helps to open the tight junction between the epithelial cells leading to enhanced permeation of THQ [60].

### 3.8. Measurement of Permeation Depth

**Figure 13:**
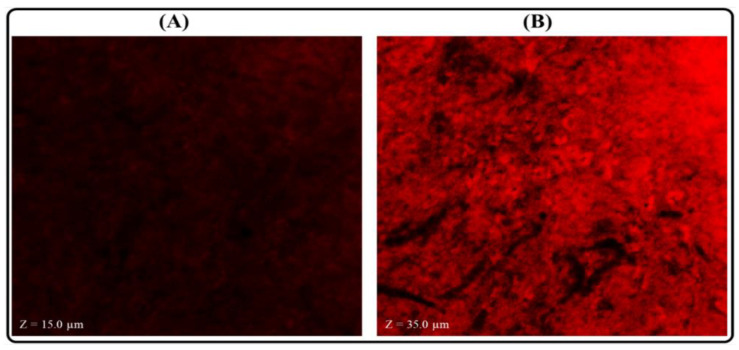
The image shows the confocal micrographs of intestinal tissue after treatment with (**A**) plain RhB solution and (**B**) RhB-LPHNPs.

This investigation was conducted to analyze the ability of THQ-LPHNPs to penetrate the deeper layers of the small intestine. The penetration depth was analyzed by CLSM and the comparative results of THQ-LPHNPs and plain RhB solution are represented in Figure 13. The ‘*z*-axis’ for RhB-LPHNPs and plain RhB treated slides of intestinal tissues were noted to analyze the depth of RhB that was penetrated in the layers of the small intestine. As expected, the optimized RhB-LPHNPs showed much greater penetration (z = 35.0 μm) into the layers of the small intestine in comparison with pure RhB solution (z = 15.0 μm). A higher penetration of the RhB-LPHNPs was ascribed to the small size of the LPHNPs. Further, the incorporation of CHS in the nanocarrier opens the tight junction between the epithelial cells which further enhances the penetration in the layers of small intestine [61]. Therefore, confocal microscopy further confirmed the higher intestinal absorption of THQ-LPHNPs in comparison to plain RhB solution.

### 3.9. Relative Bioavailability Study

**Figure 14:**
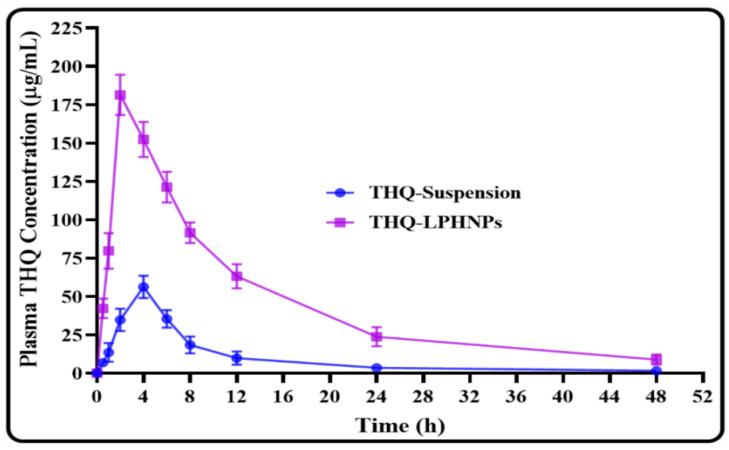
Image representing the plasma THQ concentration vs. time profiles after a single dose oral administration of THQ-LPHNPs and the conventional THQ suspension. Data represented as mean ± SD, *n* = 6.

The oral bioavailability of THQ was assessed after a single dose administration of LPHNPs in Wistar rats. The results were compared with the conventional THQ suspension and the plasma profiles as well as biopharmaceutical attributes are depicted in Figure 14 and Table 6. The results of the present investigation revealed a much better biopharmaceutical performance for the optimized THQ-LPHNPs. It showed a significantly higher plasma concentration at each time point in comparison with the conventional THQ suspension. It showed much faster drug absorption and achieved peak plasma concentration within 2 h. Whereas, the conventional THQ suspension takes 4 h to reach the peak plasma concentration. THQ-LPHNPs depicted the ${\mathrm{AUC}}_{0\to 48}$ and $C_{\max}$ of 2213.81 µ·h/mL and 181.49 µg/mL, respectively. While the conventional THQ suspension showed the ${\mathrm{AUC}}_{0\to 48}$ and $C_{\max}$ of 466.215 µ·h/mL and 56.23 µg/mL, respectively. Thus, THQ-LPHNPs exhibited 4.74 and 3.22 folds higher (*p* < 0.05) oral bioavailability and $C_{\max}$ compared to the conventional THQ suspension. Further, $T_{\max}$, MRT, and $t_{1/2}$, was also calculated and the optimized THQ-LPHNPs displayed 2 h, 12.14 h, and 12.77 h, respectively. On the other hand, the conventional THQ suspension exhibited the $T_{\max}$, MRT, and $t_{1/2}$ of 4 h, 10.36 h, and 9.65 h, respectively. Thus, the optimized THQ-LPHNPs showed significantly faster absorption from the intestine, enhanced systemic circulation time and plasma half-life residence time after oral administration in comparison with the conventional THQ suspension. The enhancement in the absorption and plasma concentration with THQ-LPHNPs was achieved due to the small particle size of LPHNPs which provides a much higher surface area for absorption from the small intestine. In addition, the encapsulation of THQ in the LPHNPs in the amorphous form significantly enhances the solubility of THQ [62,63]. Moreover, the excellent mucoadhesive properties of THQ-LPHNPs further contribute to the greater absorption of THQ from the small intestine. The positively charged mucoadhesive THQ-LPHNPs interact with the negatively charged mucous membrane, enhancing the nanocarrier’s residence in the small intestine [64,65]. On the other hand, the poor biopharmaceutical attributes of the conventional THQ suspension were ascribed to the poor aqueous solubility of THQ in the gastrointestinal fluids.

## 4. Conclusions

This investigation was based on the development of THQ-LPHNPs by the nanoprecipitation method. The optimized THQ-LPHNPs revealed excellent characteristics for oral delivery of highly lipophilic compounds (THQ). It showed a nano-metric size (179.63 ± 4.77 nm), a positive high zeta potential (>25 mV), and a low PDI value (0.217 ± 0.013). It also showed high %EE and %DL due to a hybrid matrix of LPHNPs. Initially, a fast THQ release was achieved for 4 h, after which a sustained release was found for up to 48 h. The natural polymer CHS provides significantly higher mucoadhesive properties to LPHNPs that help to enhance intestinal permeation and lead to enhanced bioavailability after oral administration. THQ-LPHNPs demonstrated 4.7 times greater bioavailability than THQ suspension. It revealed higher cytotoxicity against MDA-MB-231 and MCF-7 cells in comparison with free THQ. Thus, formulating mucoadhesive LPHNPs might offer a new opportunity to enhance oral bioavailability as well as therapeutic efficacy of lipophilic phytochemicals such as THQ for the management of different solid tumors.

## Figures and Tables

**Table 1:** Selected independent and dependent variables used in the Box–Behnken design to develop THQ-LPHNPs.

Factors	Levels		
Independent variables	Low (−1)	Medium (0)	High (+1)
F_1_ = PL-90G concentration (mg)	100	125	150
F_2_ = CHS concentration (mg)	50	70	90
F_3_ = P-188 concentration (mg)	75	100	125
Responses (Dependent variables)	Goal		
R_1_ = Particle size (PS; nm)	Minimize		
R_2_ = Polydispersity index (PDI)	Minimize		
R_3_ = Entrapment efficiency (EE; %)	Maximize		

**Table 2:** Box–Behnken design experimental runs observed for the development of THQ-LPHNPs with their actual and predicted experimental values of R_1_ (PS), R_2_ (PDI), and R_3_ (%EE).

Runs	Independent Factors			Dependent Factors (Responses)					
	F_1_ (mg)	F_2_ (mg)	F_3_ (mg)	R_1_ (PS in nm)		R_2_ (PDI)		R_3_ (EE in %)	
				Actual	Predicted	Actual	Predicted	Actual	Predicted
NP1	100	50	100	123.79	121.10	0.126	0.118	69.86	69.99
NP2	125	50	125	152.26	154.67	0.181	0.179	79.78	79.67
NP3	150	70	125	214.19	212.81	0.352	0.348	93.27	93.30
NP4	125	90	125	203.48	204.18	0.327	0.323	90.72	90.83
NP5	125	70	100	180.52	183.58	0.219	0.222	84.37	84.48
NP6	150	70	75	227.67	224.39	0.372	0.363	90.53	90.55
NP7	100	70	125	142.78	143.06	0.159	0.167	77.61	77.59
NP8	150	50	100	194.47	195.44	0.271	0.275	85.79	85.88
NP9	125	50	75	165.09	164.39	0.205	0.208	76.37	76.26
NP10	125	70	100	181.14	180.58	0.224	0.222	84.71	84.48
NP11	100	70	75	149.85	151.24	0.183	0.186	73.76	73.73
NP12	125	70	100	180.09	180.58	0.225	0.222	84.37	84.48
NP13	125	90	75	215.63	215.22	0.328	0.329	87.52	87.63
NP14	150	90	100	246.53	247.22	0.423	0.430	97.65	97.52
NP15	100	90	100	174.63	173.66	0.233	0.228	80.97	80.88

**Table 3:** Results of regression analysis for all three responses i.e., R_1_ (PS in nm), R_2_ (PDI), and R_3_ (EE in %) after fitting the data into different models.

Model	R^2^	Adjusted R^2^	Predicted R^2^	SD	Press	Remark
Response-1 (R_1_)						
Linear	0.9967	0.9958	0.9944	2.21	89.93	-
2F1	0.9973	0.9953	0.9924	2.32	122.58	-
Quadratic	0.9995	0.9985	0.9919	1.32	131.55	Suggested
Response-2 (R_2_)						
Linear	0.9518	0.9387	0.9272	0.0214	0.0076	-
2F1	0.9580	0.9265	0.9037	0.0235	0.0101	-
Quadratic						Suggested
Response-3 (R_3_)						
Linear	0.9970	0.9962	0.9953	0.4684	3.78	-
2F1	0.9976	0.9958	0.9944	0.4942	4.55	-
Quadratic	0.9998	0.9994	0.9978	0.1887	1.79	Suggested

**Table 4:** Stability of THQ-LPHNPs in SGF (pH 1.2) and SIF (pH 6.8). *n* = 3, mean ± SD.

Parameters	SGF (pH = 1.2)		SIF (pH = 6.8)	
	Initial	Final	Initial	Final
Particles size (nm)	179.63 ± 4.77	193.31 ± 6.44	179.63 ± 4.77	187.82 ± 5.39
Polydispersity index	0.217 ± 0.013	0.272 ± 0.016	0.217 ± 0.013	0.241 ± 0.013
Entrapment efficiency (%)	85.49 ± 3.73	78.54 ± 3.57	85.49 ± 3.73	81.46 ± 3.39
Zeta potential (mV)	+26.52 ± 2.21	+21.18 ± 2.83	+26.52 ± 2.21	+23.53 ± 1.94

**Table 5:** The results of different release kinetics models for THQ-LPHNPs.

Model	Model Equation	Equation	R^2^	Release Exponent (‘*n*’)
Zero-order model	M_t_ = M_0_ + k_0_ t	y = 0.0127x + 0.3162	0.5585	-
First-order model	ln M_t_ = ln M_0_ + k_1_ t	y = –0.0124x + 1.8232	0.7214	-
Higuchi-matrix model	M_t_ = M_0_ + k t^1/2^	y = 0.1162x + 0.1339	0.7824	-
Korsmeyer–Peppas model	M_t_/M_∞_ = k t^n^	y = 0.5522x − 0.8337	0.8736	0.2397

**Table 6:** Pharmacokinetic parameters of THQ-LPHNPs and conventional THQ suspension after a single dose administration in Wistar Albino rats (*n* = 6).

Parameters	THQ-Suspension	THQ-LPHNPs
$C_{\max}$ (µg/mL)	56.23	181.49 *
$T_{\max}$ (h)	4	2
${\mathrm{AUC}}_{0\to 48}$ (µ·h/mL)	466.215	2213.807 *
${\mathrm{AUC}}_{0\to \infty }$ (µ·h/mL)	492.789	2376.788 *
${\mathrm{AUMC}}_{0\to 48}$ (µ·h^2^/mL)	4830.007	26897.355 *
${\mathrm{AUMC}}_{0\to \infty }$ (µ·h^2^/mL)	6552.578	37725.244 *
MRT (h)	10.360	12.149
$t_{1/2}$ (h)	9.658	12.779 *
$K_{\mathrm{el}}$ (h^−1^)	0.059	0.047
$F_{\mathrm{rel}}$	–	4.748

* Denotes significantly (*p* < 0.05) different values of the optimized THQ-LPHNPs compared to THQ-suspension.

## Data Availability

Not applicable.
